# Rank orders of mammalian pathogenicity-related PB2 mutations of avian influenza A viruses

**DOI:** 10.1038/s41598-020-62036-5

**Published:** 2020-03-24

**Authors:** Chung-Young Lee, Se-Hee An, Jun-Gu Choi, Youn-Jeong Lee, Jae-Hong Kim, Hyuk-Joon Kwon

**Affiliations:** 1 Laboratory of Avian Diseases, College of Veterinary Medicine, Seoul National University, 08826 Seoul, Republic of Korea; 2 Department of Farm Animal Medicine, College of Veterinary Medicine, Seoul National University, 08826 Seoul, Republic of Korea; 3 Research Institute for Veterinary Science, College of Veterinary Medicine, Seoul National University, 08826 Seoul, Republic of Korea; 4 Avian Disease Division, Animal and Plant Quarantine Agency, 177, Hyeoksin 8-ro, Gyeongsangbuk-do, 39660 Republic of Korea; 5 Farm Animal Clinical Training and Research Center (FACTRC), GBST, Seoul National University, Kangwon-do, Republic of Korea

**Keywords:** Viral pathogenesis, Influenza virus, Infection

## Abstract

The PB2 gene is one of the key determinants for the mammalian adaptation of avian influenza A viruses (IAVs). Although mammalian pathogenicity-related mutations (MPMs) in PB2 genes were identified in different genetic backgrounds of avian IAVs, the relative effects of single or multiple mutations on viral fitness could not be directly compared. Furthermore, their mutational steps during mammalian adaptation had been unclear. In this study, we collectively compared the effects of individual and combined MPMs on viral fitness and determined their rank orders using a prototypic PB2 gene. Early acquired mutations may determine the function and potency of subsequent mutations and be important for recruiting multiple, competent combinations of MPMs. Higher mammalian pathogenicity was acquired with the greater accumulation of MPMs. Thus, the rank orders and the prototypic PB2 gene may be useful for predicting the present and future risks of PB2 genes of avian and mammalian IAVs.

## Introduction

Waterfowl are reservoirs for influenza A viruses (IAVs), and close interaction between waterfowl and other animals causes occasional cross species transmission to result in successful settle-down by acquiring host adaptive mutations in their eight segmented genomes (PB2, PB1, PA, HA, NP, NA, M, and NS)^1^. In particular, the PB2 protein, which is involved in cap snatching of the host mRNA, has been regarded as one of the key molecules to overcome species-specific host barriers^2–4^. PB2 interacts with various host proteins, including importin-α for nuclear localization and ANP32A for polymerase stabilization and mitochondrial antiviral signaling proteins (MAVS), Tu elongation factor, and mitochondrial (TUFM) for anti-viral activity^5–8^.

To date, many mammalian pathogenicity-related mutations (MPMs, D9N, I147T, E158G, E192K, A199S, D253N, T271A, K339T, F404L, K526R, A588I, A588T, G590S, Q591R, Q591K, E627K, A674T, D701N, K702R, and S714R, etc.) in PB2 have been reported in different domains of the PB2 protein^3,4,9–27^. The E627K mutation most potently increases polymerase activity, replication efficiency and pathogenicity of avian IAVs in mammalian hosts^3,4,17^. However, avian IAVs cumulatively have acquired multiple mutations other than E627K, and there may be additional requirements to achieve successful adaptation to mammals. As MPMs were identified and verified in different genetic contexts, the relative effects of MPMs on viral fitness needed to be compared in the same genetic backgrounds to omit epistasis^28^. In addition, swine-origin pandemic H1N1 viruses in 2009 [A(H1N1) pdm2009] showed human pathogenicity without E627K mutation, and different mutational steps to acquire mammalian pathogenicity were proposed^21^. Human and swine IAVs possess already multiple MPMs, but their effects on mammalian pathogenicity have never been compared collectively. Furthermore, the mutational steps of MPMs during mammalian adaptation were unclear.

Previously, we found a prototypic avian PB2 gene (01310 PB2) that completely attenuated PR8-derived recombinant virus (7 + 1 reassortant) in mice and did not possess any MPMs except 661A and 683T, and identified the minimum essential mutations (I66M, I109V, and I133V, MVV mutations) to acquire additional MPMs such as Q591R/K, E627K, and D701N^29,30^. The minimum essential mutations increased viral replication efficiency in both avian and mammalian cells and were postulated to increase the trimeric integrity of polymerase complex^29^. As epistatic mutations blur the effects of certain mutations, the use of the prototypic PB2 gene may be crucial to minimize epistasis^28^.

Therefore, in this study, we grafted each of 20 MPMs of 18 amino acid residues related to mammalian pathogenicity in the 01310PB2 gene and compared their effects on polymerase activity. Then, we generated PR8-derived recombinant viruses with selected mutations in 01310PB2 to compare replication efficiency in mammalian hosts. According to the modified median-joining network analysis based on profiles of the twenty MPMs and chronological data, we hypothesized mutational steps of MPMs in major PB2 genotypes of A(H1N1) pdm2009 and seasonal flu viruses. We compared polymerase activities of the hypothetical intermediate and final combinations of MPMs acquired during adaptation to pigs and humans and generated PR8-derived recombinant viruses possessing major combinations of MPMs to compare their effects on viral replication efficiency and pathogenicity in mammalian hosts.

## Results

### The frequency of each MPM in different categories of PB2 genes

**Figure 1:**
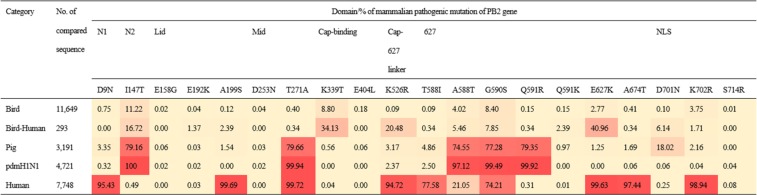
Mammalian pathogenic PB2 mutations and frequency in different hosts. The location and frequency of 20 MPMs of 18 amino acid residues of PB2 were compared and the frequency difference was visualized by heat-map, 0 (yellow) to 100% (red).

We collected 27,602 PB2 genes possessing MVV mutations that comprised 84.7% of PB2 genes (32,591) in the Influenza Research Database (IRD)^29^. We classified them into 5 categories, Bird (avian IAVs), Bird-Human [avian IAVs isolated form humans (H5, H6, H7, H9, and H10 IAVs)], Pig (swine IAVs), pdmH1N1 (H1N1 IAVs during 2009 pandemic), and Human (human IAVs), and calculated the frequencies of their MPMs. The twenty mutations of 18 amino acid residues, D9N, I147T, E158G, E192K, A199S, D253N, T271A, K339T, F404L, K526R, A588I/T, G590S, Q591R/K, E627K, A674T, D701N, K702R, and S714R, were located in different domains of PB2 and the frequency difference was visualized by heat map (Fig. 1).

The frequency of each mutation was apparently different between different categories. The frequencies of the mutations D9N, A199S, K526R, A588I, E627K, A674T, and K702R were highest in Human relative to the other categories, but the frequencies of mutations I147T, A588T, and Q591R were higher in the Pig and pdmH1N1 categories than in the others. Although the frequency of D701N in Pig (18.02%) was not high, it was relatively higher than that in other categories (0.06–0.25%). Only T271A and G590S were commonly frequent in the Pig, pdmH1N1 and Human categories. Although most of mutations were less frequent in Bird than in other categories, I147T, K339T, A588T, G590S, E627K, and K702R were relatively more frequent than the other mutations. In the Bird-Human category, the frequencies of E627K (from 2.77 to 40.96%), K339T (from 8.8 to 34.13%), and K526R (from 0.09 to 20.48%) increased steeply in comparison with the corresponding mutations in the Bird category. The frequencies of E158G, E192K, D253N, F404L, Q591K, and S714R were very low, less than 2.4%, in all categories.

### The effect of each MPM on polymerase activity, replication efficiency and pathogenicity in mammalian hosts

**Figure 2:**
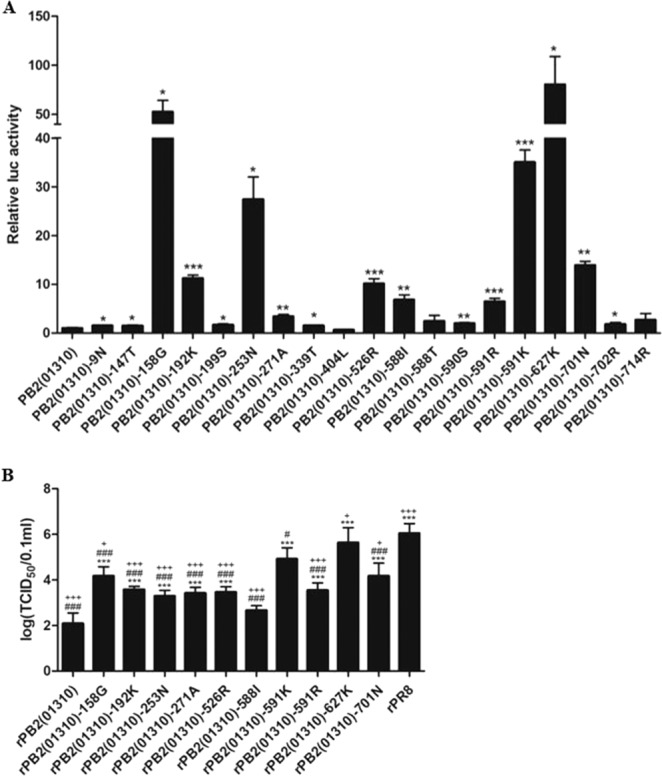
Effect of single MPMs on polymerase activity and replication efficiency in MDCK cells. Polymerase activity was measured using mini-genome assays in 293T cells **(A**). The data were normalized to the polymerase activity of the wild-type 01310 PB2 gene. Statistical significance was calculated using one-way ANOVA (^*^P < 0.05, ^**^P < 0.01, ^***^P < 0.001). Replication efficiency of PR8-derived recombinant viruses possessing single MPMs in 01310PB2 gene in MDCK cells at 37 °C (**B**). Wild-type and mutant viruses (10^7^ EID_50_/0.1 mL) were used to infect MDCK cells, and the TCID_50_ was measured at 5dpi. Statistical significance was analyzed by one-way analysis of variance with Bonferroni correction (compared to rPB2(01310), ^***^P < 0.001; compared to rPB2(01310)-627K, ^#^*P* < 0.05, ^###^*P* < 0.001; compared to rPB2(01310^)^-591K, ^+^*P* < 0.05, ^+++^*P* < 0.001). The data are the average of triplicate data ± s.d. from one experiment.

To compare the effect of each PB2 mutation on polymerase activity, we selected 19 MPMs except A674T, which had no effect on polymerase activity^29^. We measured the polymerase activity by using an *in vitro* mini-genome assay in 293T cells (Fig. 2A). Except for the F404L, A588T, and S714R mutations, all PB2 mutations significantly increased polymerase activity relative to the wild-type 01310 PB2 gene. In particular, the E627K, E158G, Q591K, and D253N mutations showed the highest polymerase activities in order and increased polymerase activity by more than 20-fold in comparison with that of the 01310 PB2 gene (Fig. 2A). D701N, E192K and K526R showed approximately 10-fold increases in polymerase activities, followed by A588I and Q591R. Although D9N, I147T, A199S, T271A, K339T, G590S, and K702R showed significantly higher polymerase activity than wild-type 01310 PB2, they increased polymerase activities by 3-fold or less. The rank order of the effects of PB2 mutations on polymerase activity was summarized in Table S2.

To investigate the effects of the MPMs on viral replication, we selected 10 mutations that increased the polymerase activity by more than 3-fold (E158G, E192K, D253N, T271A, K526R, T588I, Q591K, Q591R, E627K, and D701N) and generated PR8-derived recombinant viruses containing the 10 mutated 01310 PB2 genes. In MDCK cells, most of the mutant viruses showed significantly higher replication efficiency than the wild-type virus, rPB2(01310), except rPB2(01310)-588I (P < 0.05) (Fig. 2B). The E627K mutation increased virus titers significantly in comparison with other mutations. The Q591K mutation more greatly increased virus titer than the E158G and D701N mutations (Fig. 2B). The rank order of the effects of MPMs on replication efficiency in MDCK cells was summarized in Table S2.

**Figure 3:**
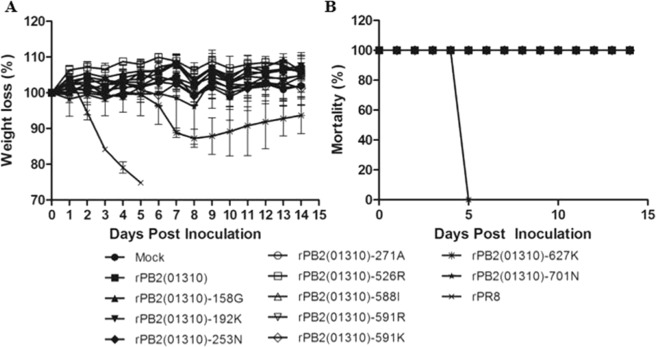
Effects of single MPMs on mouse pathogenicity. The pathogenicity of single amino acid mutant viruses was observed by body weight loss (**A**) and mortality (**B**) of infected mice. Five 6-week-old BALB/c mice were challenged with 1.0 × 10^6^ EID_50_ of each virus or PBS (mock). Weight loss was observed for 14 days, and mice showing more than 20% body weight loss were regarded as dead. The average weight loss ± s.d. was measured by comparing to the initial weight of each mouse.

We investigated the effect of the 10 PB2 mutations on mouse pathogenicity by observing body-weight loss of BALB/c mice (Fig. 3). Most of the single mutations introduced into the prototypic 01310 PB2 gene did not cause body-weight loss during the observation period, but rPB2(01310)-E627K slightly induced the body weight loss of mice up to 12% (Fig. 3A). Only rPR8 caused mortality during the observation period (Fig. 3B). However, all of the single amino acid mutant viruses could replicate in the lungs on 3 and/or 6 dpi in contrast to the parent virus, rPB2(01310) (Table 1). As expected, rPB2(01310)-627K showed the highest virus titers among other viruses on 3 dpi, but rPB2(01310)-701N reached to the highest virus titers on 6 dpi. The effects of E158G, 192K, 271A and Q591K on virus replication in the lungs were less apparent than those of E627K and D701N but higher than those of D253N, A588I and Q591R. The rank order of the effects of MPMs on virus replication in the lungs was summarized in Table S2.

**Table 1:** Comparison of the viral replication efficiency of 01310 PB2 single amino acid mutant viruses in mouse lungs.

Virus	3 dpi		6 dpi	
	Positive rate^*^	Virus titer (logEID_50_/0.1 mL)	Positive rate	Virus titer (logEID_50_/0.1 mL)
Mock (PBS)	0/3	<0.5^†^	0/3	<0.5
rPB2(01310)	0/3	<0.5	0/3	<0.5
rPB2(01310)-158G	3/3	3.2 ± 1.7	3/3	3.1 ± 0.9
rPB2(01310)-192K	3/3	2.5 ± 1.3	3/3	2.8 ± 2.1
rPB2(01310)-253N	0/3	<0.5	1/3	0.8 ± 0.6
rPB2(01310)-271A	3/3	2.4 ± 0.5	3/3	3.5 ± 1.5
rPB2(01310)-526R	2/3	1.3 ± 1.4	2/3	2.3 ± 1.5
rPB2(01310)-588I	1/3	1.1 ± 1.0	1/3	1.5 ± 1.7
rPB2(01310)-591R	2/3	<0.5	3/3	1.5 ± 1.1
rPB2(01310)-591K	3/3	2.9 ± 1.2	3/3	3.4 ± 0.4
rPB2(01310)-627K	3/3	5.0 ± 0.4	3/3	4.4 ± 0.4
rPB2(01310)-701N	3/3	3.8 ± 0.9	3/3	4.8 ± 0.3
rPR8	3/3	6.1 ± 0.5	1/1	5.0^‡^

*Number of positive samples/number of mice (virus titer of the pooled lung tissues, log10 EID_50_/0.1 mL).

^†^Below the limit of assay detection.

^‡^Data from only one mouse due to the death of two mice.

### Inference of the evolutionary steps of swine and human PB2 genes

**Figure 4:**
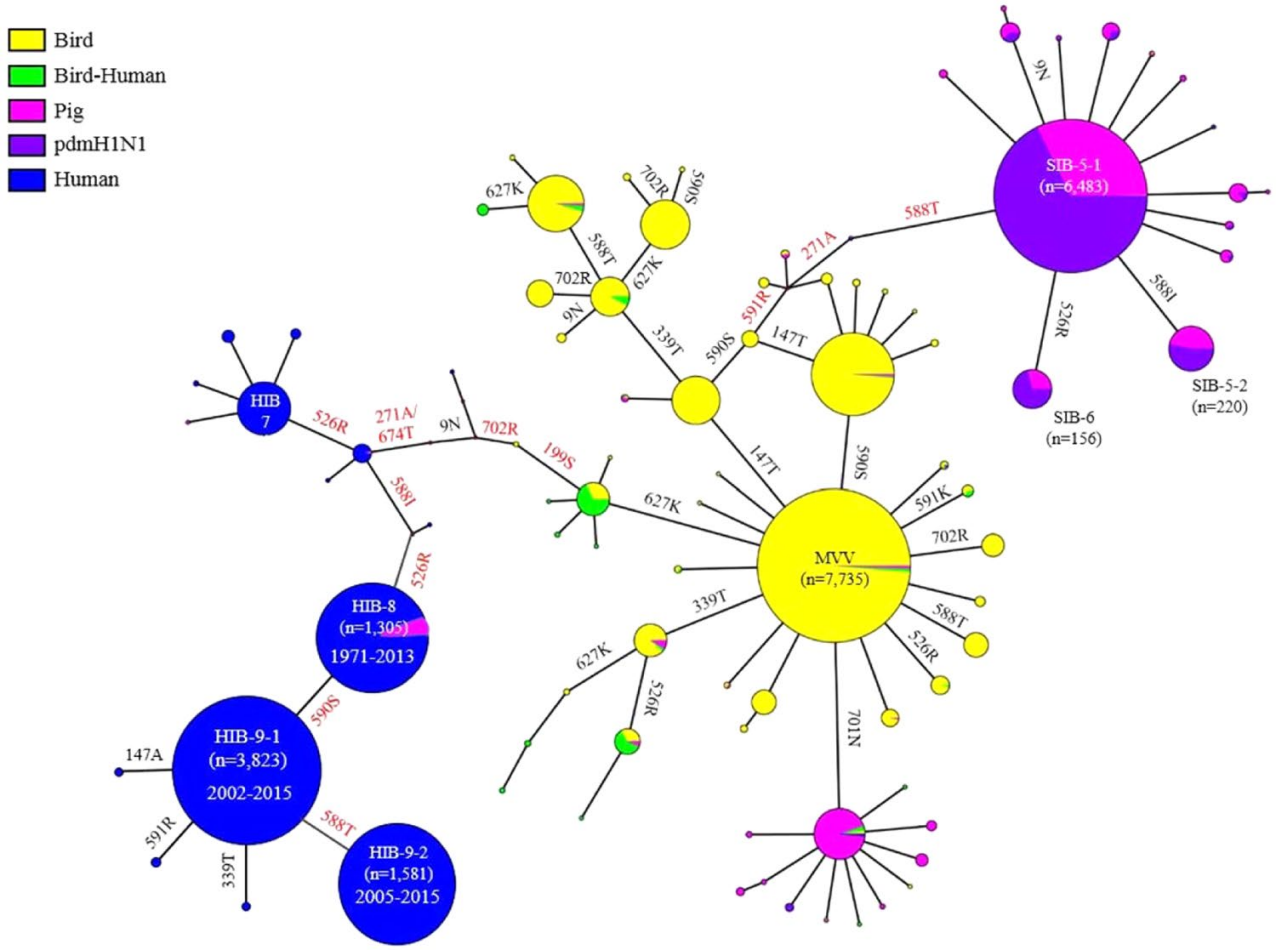
Phylogenetic network analysis of avian, swine and human PB2 genes and mutational steps of MPMs during mammalian adaptation. The profiles of MPMs of PB2 genes (n = 24,825) were analyzed by median joining network analysis with Network 5.0.0. Major genotypes were represented in the circles, and the size of circle is proportional to its frequency. Five different categories were represented in different colors. According to the manual chronology and frequency analysis of MPMs, mutational steps of major genotypes (SIB-5-1, SIB-5-2, SIB-6, HIB-8, HIB-9-1, and HIB-9-2) were hypothesized and represented in red color letters.

To understand the mutational steps of MPMs, we performed median joining network analysis using the profiles of 20 mutations of PB2 genes. According to the results, predominant PB2 genotypes in avian, A(H1N1) pdm2009, and human seasonal flu viruses were apparent: MVV, SIB-5–1 and HIB-9-1, respectively. The avian MVV genotype may have evolved to become SIB-5-1 and HIB-9-1 through multiple mutational steps (Fig. 4). However, the chronological data of the PB2 genes were not reflected in the median joining network analysis tree. Therefore, we modified the order of mutational steps from MVV to SIB-5-1 and HIB-9-1 based on chronology-frequency consideration of PB2 mutations as described below.

In the case of the SIB-5-1 genotype, avian IAVs possessing MVV-G590S, MVV-I147T, and MVV-G590S-I147T were first observed in 1977 [A/canvasback_duck/ALB/274/1977 (H4N6), CY004728)], 1968 [A/turkey/Wisconsin/1/1968 (H5N9), CY080514] and 1986 [A/ blue_winged_teal/LA/B228/1986 (H1N1), EU743313], respectively. Therefore, early PB2 genes bearing MVV-G590S and MVV-I147T have coexisted, and MVV-G590S-I147T may have appeared after acquiring the I147T or G590S mutation (Fig. 4). Considering the relatively low frequency and absence of MVV-G590S-I147T among avian and mammalian IAVs, respectively (relatively small size and yellow circle), it may have acquired additional Q591R, T271A and A588T successively due to the very low frequencies of the intermediates (MVV-G590S-I147T-Q591R, MVV-G590S-I147T-Q591R-T271A and MVV-G590S-I147T-Q591R-T271A-A588T) of MVV-G590S-I147T and SIB-5-1 (Fig. 4). Most A(H1N1) pdm2009 viruses possessed both Q591R and T271A, but only two strains possessed either Q591R or T271A. Therefore, MVV-G590S-I147T may have acquired Q591R and T271A mutations nearly simultaneously. After the following acquisition of A588T, SIB-5-1 may have become predominant, and evolved to become SIB-5-2 or SIB-6 by acquisition of T588I or K526R. Therefore, PB2 genes of A(H1N1) pdm2009 viruses may have acquired G590S/I147T, Q591R/T271A, A588T, T588I, and K526R mutations cumulatively (Fig. 4).

In the case of HIB-9-1 the PB2 gene of A(H1N1) pdm1918 already possessed MVV, A199S, E627K and K702R, and we inferred firstly the cumulative order of the A199S, E627K and K702R mutations (Table S3). The E627K mutation may have been acquired earlier due to an apparent higher frequency than that of A199S (Fig. 1). In the case of swine PB2 genes with TIV instead of MVV, E627K-A199S were observed without K702R and D9N, and A199S may precede K702R and D9N (Table S4). However, whether K702R or D9N was acquired next to A199S was hard to determine because swine PB2 genes with TVV instead of MVV during the 1930s had K702R without D9N, while in contrast, swine TIV PB2 in the 1970s only had D9N without K702R (Table S5). However, A/Brevig Mission/1/1918 (H1N1) possesses only K702R without D9N, and we represented K702R as earlier than D9N (Table S1, Fig. 4). Out of the second earliest human H1N1 viruses during 1933-1936 (8 strains), five strains possessed K702R without T271A, and two strains have both T271A and K702R. Only the PR8 strain (4 out of 5 registered PR8 sequences in the database) possesses T271A without K702R. Therefore, K702R may precede T271A. In addition, five strains during 1933-1936 possess A674T without T271A, with 3 strains possessing both. Therefore, A674T may precede T271A (Table S3). The PB2 of A(H3N2) pdm1968 viruses possessed both T271A and A588I mutations, and it could not be determined which one was acquired earlier. Additionally, single point mutations do not cause A to I but do cause V to I mutations at 588 residue. Therefore, higher frequency of 588V than 588A in the incomplete mutant PB2 may support V588I mutation (Table S6). The HIB-8 genotype might have acquired K526R after the T271A and V588I mutations (Fig. 4, Table S7). The HIB-8 genotype was prevalent among seasonal flu viruses during 1971-2013, but the HIB-9-1 and HIB-9-2 genotypes were prevalent during 2002-2015 and 2005-2015, respectively. HIB-9-1 might have evolved from HIB-8 by acquisition of G590S and HIB-9-2 from HIB-9-1 by acquisition of the I588T mutation (Fig. 4). HIB-9-1 and HIB-9-2 still evolved to acquire additional MPMs such as I147T, K339T, Q591R or D701N (Table S8). Therefore, MVV PB2 genes may have acquired E627K, A199S, K702R, D9N, A674T, T271A/V588I, K526R, G590S, and I588T cumulatively (Fig. 4, Table S1). Although the predominant SIB- and HIB-series of PB2 genotypes started from different early mutations (MVV-G590S/I147T and MVV-E627K), the last half of the accumulation steps of SIB- and HIB-series genotypes shared similar mutations: T271A, A/V588T/I, and K526R. In addition, HIB-9-1 genotype PB2 genes have evolved by acquiring additional mutations such as I147T, Q591R, or K339T (Fig. 4).

### The effects of combined multiple MPMs on polymerase activity

**Figure 5:**
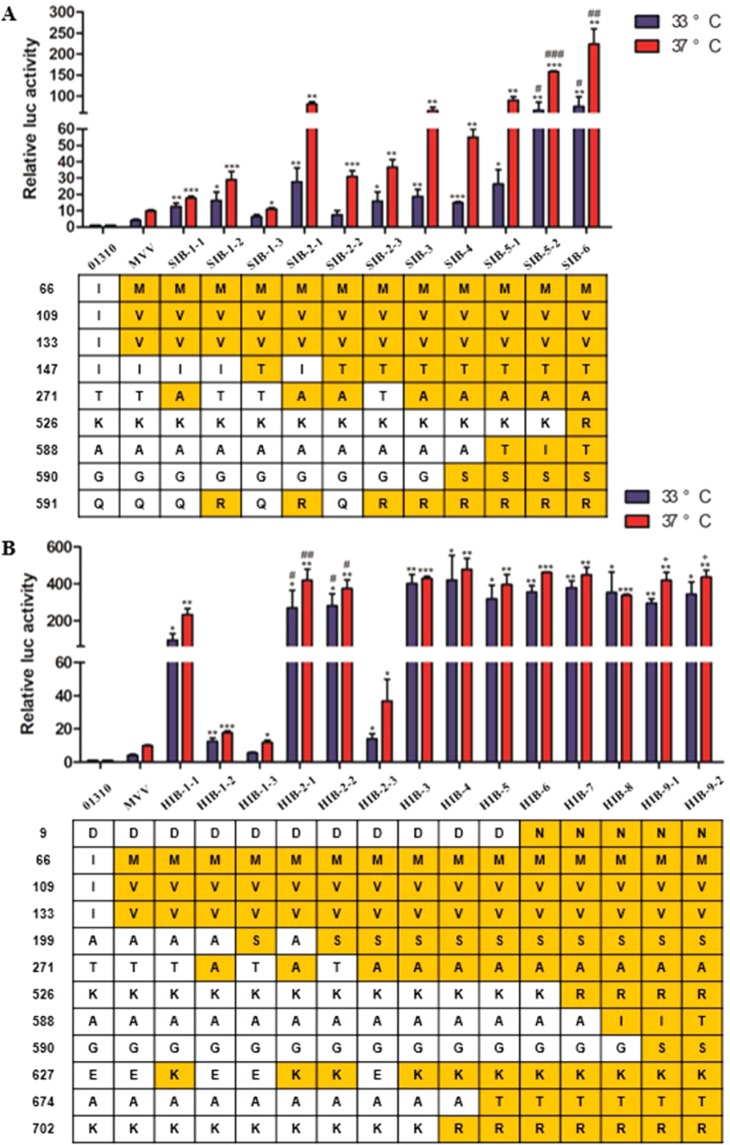
The effect of combined multiple MPMs on polymerase activity. Polymerase activity was measured using mini-genome assays in 293T cells at 33 and 37 °C. The data were normalized to the polymerase activity of the wild-type 01310 PB2 gene. Statistical significance was calculated using one-way ANOVA. Panel A, Polymerase activity of each combined MPM of the SIB-series (compared to PB2(01310)-MVV, ^*^P < 0.05, ^**^P < 0.01, and ^***^P < 0.001; compared to SIB-5-1, ^#^*P* < 0.05, ^##^*P* < 0.01, ^###^*P* < 0.001)^.^ The data are the average of three independent experiments ± s.d. Panel B, Polymerase activity of each combined MPMs of the HIB-series (compared to PB2(01310)-MVV, ^*^P < 0.05, ^**^P < 0.01, and ^***^P < 0.001; compared to HIB-1-1, ^#^*P* < 0.05, ^##^*P* < 0.01; compared to HIB-8, ^+^ *P* < 0.05). The data are the average of three independent experiments ± s.d.

Previously, we found that the introduction of MVV mutations to prototypic 01310PB2 led to the acquisition of additional mutations such as Q591K, Q591R, E627K, and D701N in the lungs of BALB/c mice^29^. Moreover, the Q591K, Q591R, E627K, and D701N mutations are key factors for AIVs to overcome the host barrier^20–22,31–35^. Therefore, we compared the collaborative effects of MVV mutation with each Q591K, Q591R, E627K, and D701N mutation on polymerase activity. In combination with the MVV mutation, Q591K, Q591R, E627K, and D701N each showed increased polymerase activity at 37 °C, and MVV mutation had a synergistic relationship with each tested mutation (Fig. S1). The polymerase activity of MVV-E627K was highest, followed by MVV-Q591K, MVV-D701N, and MVV-Q591R. In addition, the single mutation of I147T (SIB-1-3) and A199S (HIB-1-3) significantly increased polymerase activity only at 37 °C, but T271A (SIB-1-1) increased polymerase activity at both 33 °C and 37 °C (Fig. 5). The polymerase activity of I147T (SIB-1-3) and A199S (HIB-1-3) was similar to each other, but the polymerase activity of T271A (SIB-1-1) was higher than that of I147T (SIB-1-3) and A199S (HIB-1-3) and lower than that of Q591R (SIB-1-2).

The synergistic effect of T271A and Q591R (SIB-2-1) apparently caused a significant increase in polymerase activity at 37 °C in comparison with two other pairs, T271A-I147T (SIB-2-2) and Q591R-I147T (SIB-2-3). Although SIB-3 (I147T), SIB-4 (I147T-A590S) and SIB-5 (I147T-A590S-A588T) possessed additional mutations, their polymerase activities did not show significant differences from that of SIB-2-1. However, acquisition of the A588I and K526R mutations by SIB-5-2 and SIB-6 significantly increased their polymerase activity at 33 °C in comparison with that of SIB-5-1.

The double mutations of the HIB-2-3 genotype, A271T and A199S, increased polymerase activity at 37 °C by approximately 2-fold in comparison with the HIB-1-3 and HIB-1-2 genotypes. The single E627K mutation of the HIB-2-3 genotype led to significantly higher polymerase activity than HIB-1-1 with double mutations (A199S and T271A). Additional A199S and/or T271A mutations to E627K in the HIB-2-2, HIB-2-1 and HIB-3 genotypes increased polymerase activity in comparison with that of the HIB-2-3 genotype, and these findings suggest the 199S and 271A mutations have synergistic cooperation with the E627K mutation. However, there was no significant difference in polymerase activity among HIB-3, 4, 5, 6, 7, 8, 9-1, and 9-2 at any temperature. Therefore, the effects of additional mutations such as K702R, A674T, D9N, K526R, V588I I588T, and G590S on polymerase activity were indistinguishable.

### The effects of the predominant combinations of MPMs on replication efficiency in mammalian hosts and mouse pathogenicity

**Figure 6:**
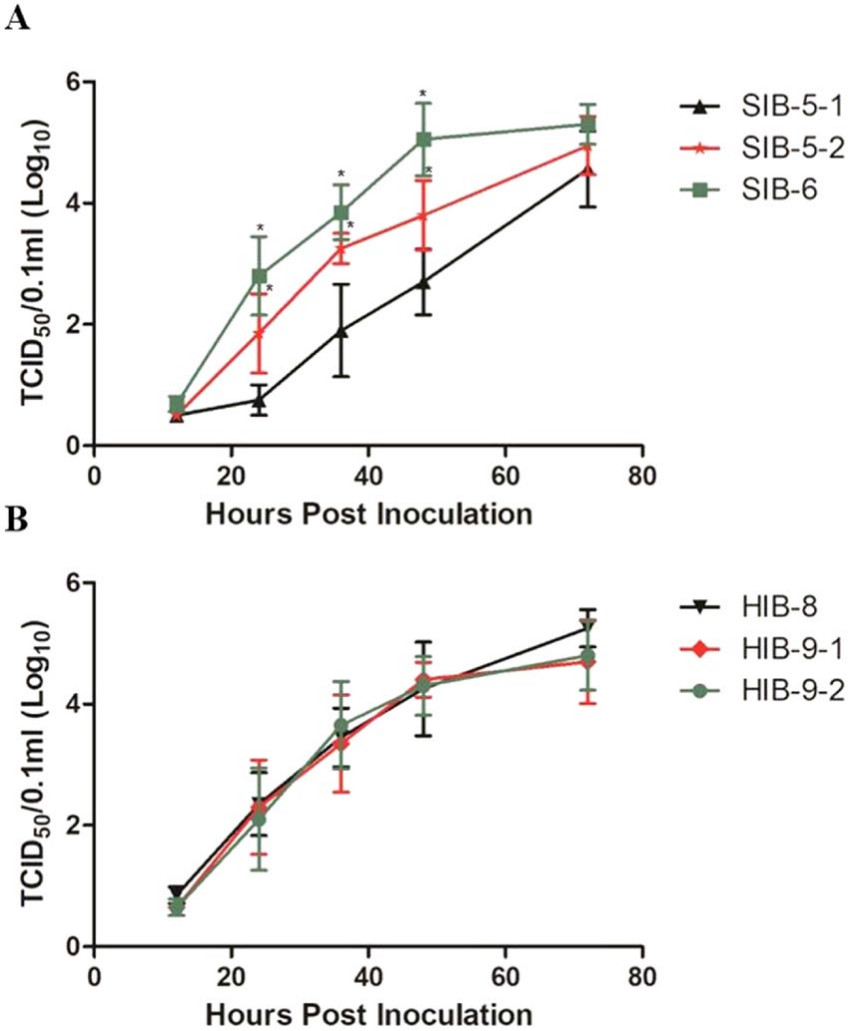
The effects of predominant swine (SIB-5-1, SIB-5-2 and SIB-6) and human (HIB-8, HIB-9-1 and HIB-9-2) PB2 genotypes on viral growth kinetics in A549 cells. The PR8-derived recombinant viruses containing major PB2 genotypes (panel A, SIB-series and panel B, HIB-series) were used to infect A549 cells (0.01 moi). The cells were incubated in a CO_2_ incubator at 37 °C, and supernatants were collected at 12, 24, 36, 48, and 72 hpi to measure viral titers in MDCK cells (TCID_50_). Statistical significance was analyzed by Student’s t test (compared to SIB-5-1, ^*^P < 0.05). The data are the average of three independent experiments ± s.d.

PR8-derived recombinant viruses containing the predominant PB2 genotypes, rPB2(01310)-SIB-5-1, rPB2(01310)-SIB-5-2, rPB2(01310)-SIB-6, rPB2(01310)-HIB-8, rPB2(01310)-HIB-9-1, and rPB2(01310)-HIB-9-2, were generated, and additionally their nucleotide sequences of PB1 and PA genes were verified to exclude the presence of co-evolutionary mutations. We compared their growth kinetics in A549 cells. rPB2(01310)-SIB-5-2 and rPB2(01310)-SIB-6 showed significantly higher replication efficiency than rPB2(01310)-SIB-5-1 at 24, 36, and 48 hpi in A549 cells (Fig. 6, P < 0.05). Therefore, the K526R and A588I mutations were involved in more efficient replication in A549 cells. However, rPB2(01310)-HIB-8, rPB2(01310)-HIB-9-1, and rPB2(01310)-HIB-9-2 showed indistinguishable replication efficiencies over all time intervals.

**Figure 7:**
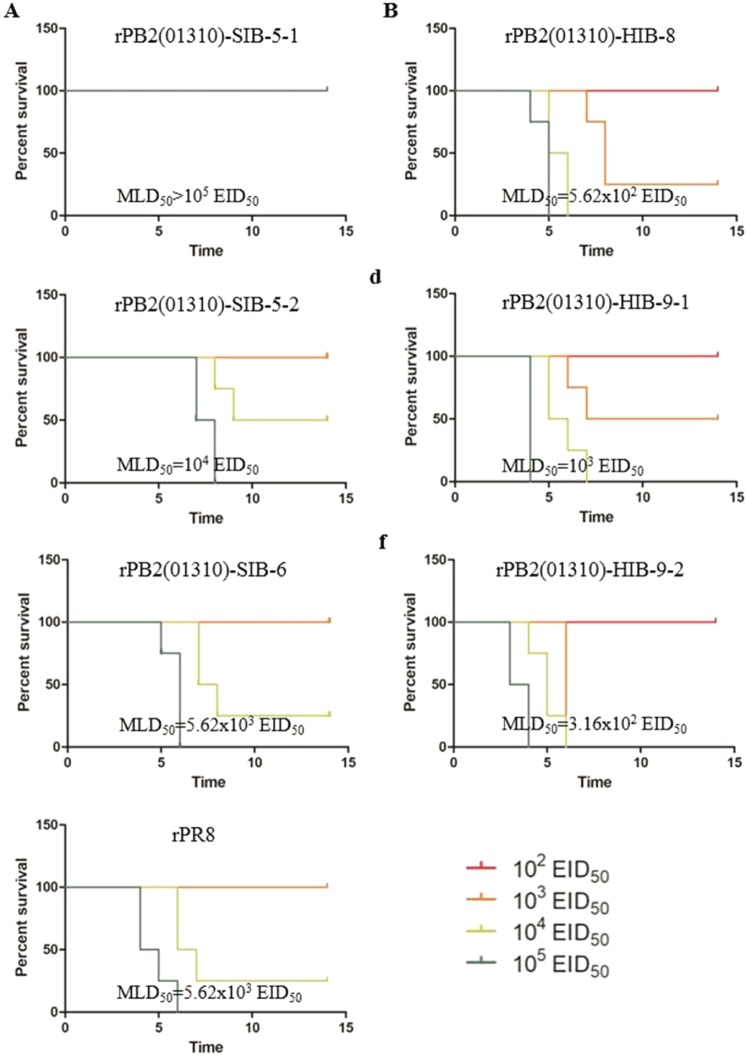
The effect of predominant swine and human PB2 genotypes on mouse pathogenicity. Six-week-old female BALB/c mice were intranasally inoculated with 10^2^ to 10^5^ EID_50_ of PR8-derived recombinant viruses containing major PB2 genotypes (panel A, SIB-series; panel B, HIB-series; panel C, rPR8), and MLD_50_ was measured. During the period up to 14 dpi, mice that lost more than 20% of their original weight were euthanized and recorded as dead.

To compare the mouse pathogenicity of the predominant PB2 genotypes, we measured the MLD_50_ by intranasal inoculation of the recombinant viruses to BALB/c mice. The MLD_50_ values of rPB2(01310)-SIB-5-1, rPB2(01310)-SIB-5-2 and rPB2(01310)-SIB-6 were >10^5^, 1.0 × 10^4^ and 5.6 × 10^3^ EID_50_, respectively. rPB2(01310)-SIB-5-2 and rPB2(01310)-SIB-6 were more pathogenic than rPB2(01310)-SIB-5-1, and rPB2(01310)-SIB-6 was comparable to rPR8 (Fig. 7). The MLD_50_ values of rPB2(01310)-HIB-8, rPB2(01310)-HIB-9-1 and rPB2(01310)-HIB-9-2 were 5.6 × 10^2^, 1.0 × 10^3^ and 3.2 × 10^2^ EID_50_, respectively. PR8-derived viruses possessing the HIB-8, 9-1 and 9-2 PB2 genotypes were more pathogenic than SIB-series and rPR8 viruses.

## Discussion

The mutation rate per site may be similar, but the effect of each mutation on viral fitness may be different ^28,36^. Mutations that increase viral fitness may become prevalent among competing mutations. During several pandemics and continuous endemics, frequent infection and transmission among humans may facilitate competition between IAVs and impose selective pressures for more competent PB2 mutations. Therefore, more than 94% frequencies of D9N, A199S, T271A, K526R, E627K, A674T, and K702R mutations in the Human category may reflect the results of intensive competition and selection of such mutations.

The earliest swine IAVs date back to 1918 and are believed to be closely related to pandemic and endemic viruses^37^. However, ‘triple reassortant’ H3N2 viruses probably appeared via reassortment of the HA, NA and PB1 genomes from human virus, NP, M and NS genomes from ‘classical’ swine virus, and PB2 and PA genomes from avian virus^38^. Then, the ‘triple reassortant’ swine descendant virus reassorted with the European avian-like porcine virus by acquiring the NA and M genomes to become A(H1N1) pdm2009 virus^39–41^. Thus, avian-origin PB2 and PA genes have co-evolved in the genetic backgrounds of swine IAVs. Although there are no pig- or human-specific mutations, higher numbers of I147T, A588T, and Q591R mutations in the Pig category than in the Human category, and different combinations of mutations, may reflect the unique mutational steps of avian-origin PB2 genes in swine^21^. As the MPM profiles of A(H1N1) pdm2009 and seasonal flu viruses are different, the early starting MPMs are likely to determine the accumulation patterns of subsequent mutations. However, the reasons why reassortant A(H1N1) pdm2009 viruses with stronger PB2 genes of seasonal flu viruses did not appear during their co-circulation may reflect importance of genetic compatibility of viral genomes^42,43^.

Most PB2 mutations tested in this study increased polymerase activity, and the rank orders of the effects on polymerase activity and replication efficiency in the mouse lungs were identical in the cases of E627K, Q591K, E192K, K526R, A588I and Q591R. However, E158G, D253N, T271A and D701N showed different rank orders, and F404L, A588T and A674T did not increase polymerase activity. Therefore, we could not exclude the positive or negative epistasis of already-present mutations of 01310PB2 (661A and 683T) or other PR8 genes such as PA and PB1 genes^30,43^. As expected, E627K is most potent mutation for the mammalian pathogenicity of IAVs, and the high ranking of E627K, D701N and Q591K was coincident with their high frequency among quasi-species during the first mouse infection of a PR8-derived recombinant virus with the 01310 PB2-MVV gene^20,29,32,33^. The 591K mutation was much more potent than 591R in terms of mammalian pathogenicity. The high ranking but extremely low frequency of E158G mutation may raise concerns about a potent, potential risk factor for avian and swine PB2 genes if they evolve to the most compatible genomic constellation^10^.

PB2 interacts with different host factors to play multiple roles. The MPMs are located in different domains and may take part in different functions. The 627 domain containing closely located E627K, Q591K/R, G590S, and A/V588T/I mutations directly interacts with ANP32A to stimulate RNA synthesis, and E627K increases interaction with the mammalian isoform of ANP32A to result in high mammalian pathogenicity^6,9,44^. TUFM is another host restriction factor differentiating 627E from 627K and impedes the replication of avian IAVs with 627E via autophagy^7^. E627K and Q591R showed negative epistasis, and no synergistic effect was observed in combination^45^. This may be the reason for the extremely low frequency of E627K together with Q591R/K PB2 genes in all categories. However, other mutations such as G590S and A/V588T/I were acquired in addition to E627K or Q591R. The E627K mutation was not selected in avian but in mammalian hosts^29^. Therefore, acquisition of mutations in the 627 domain may be crucial for avian PB2 genes not only to replicate efficiently in mammalian hosts but also to acquire additional mutations for better fitness during competition between early mammal-adapted avian-origin PB2 genes.

The NLS domain interacts with importin-α, and the second highest effect of D701N on the replication efficiency in mouse lungs may reflect the importance of the nuclear localization of PB2^8^. However, unsuccessful acquisition of multiple mutations by PB2 genes with the first D701N mutation may imply that the first strategy to steeply increase nuclear localization of PB2 may not be sustainable. In addition, a significantly higher frequency of less active 702R (98.94%) relative to 701N (0.25%) in the Human category in PB2 bearing E627K may reflect a tendency to determine subsequent mutations by preceding mutations.

The exact function of the third highest effect of E158G in the lid domain is still unclear, but two additional mutations (E192K and A199S) in the lid domain may support the important role of the domain. The N-terminal 200 amino acid residues of PB2 are involved in the interaction with RNP to transcribe and replicate viral RNAs^46^. In addition, basic amino acids in the N-terminal half of PB2, such as 124R, 142R/143R, 268R, and 331K/332R, play important roles in both the transcription and replication of vRNAs^47^. The exact function of 147T is still unclear, but its close location to 142R/143R may be noteworthy^23^. The T271A mutation in the mid domain increased the replication efficiency of IAVs at the human upper respiratory tract temperature, 33-34°C, and individual (T271A) and combined (T271A with MVV) mutations significantly increased polymerase activity at 37°C or at both 33 and 37°C, respectively, relative to 01310PB2 in this study^24,30^.

The role or function related to the highly ranked K526R mutation in the cap-627 linker domain is unclear, but the impinging position of 520-535 residues on the cap-binding site may support speculation regarding its role in viral mRNA transcription^48^. In addition, it is noteworthy that K526R showed a higher effect than the cap-binding site mutations on polymerase activity. Considering the report that K526R enhances the effects of E627K on viral replication via coordination with NEP (nuclear export protein), the role of K526R may not be as simple as has been postulated^14^. Although the polymerase activity of HIB-7 and HIB-8 was indistinguishable, the acquisition of the K526R mutation by rPB2(01310)-SIB-6 increased polymerase activity, replication efficiency in A549 cells and mouse pathogenicity in comparison with rPB2(01310)-SIB-5-1. The K526R mutation was suggested to be a mammalian adaptation marker of avian H7N9 viruses in collaboration with the E627K and D701N mutations and to play a role in the successful adaptation of H3N2 seasonal flu since approximately 1970^14^.

The T588I mutation in the PB2 genes of A(H1N1) pdm2009 viruses increased polymerase activity at 33 and 37 °C and increased mouse pathogenicity by way of probable interaction with MAVS, resulting in inhibition of IFN-β expression^11^. The A588T mutation also increased polymerase activity and mouse pathogenicity, while 588I showed significantly higher polymerase activity than 588T in this study^23^. Although HIB-9-1 and HIB-9-2 did not show dramatic differences in polymerase activity and mouse pathogenicity, rPB2(01310)-SIB-5-2, which contained 588I, showed higher viral replication and pathogenicity in mammals than rPB2(01310)-SIB-5-1. The high frequency of HIB-9-1 but recent dominance of HIB-9-2 may reflect the fine-tuning of mutation potency for viral fitness (I588T) as observed in mutational selection between 591K and 591R or 701N and 702R (Fig. 4). Interestingly, E627K was incompatible with the PB2 gene of A(H1N1) pdm2009, but most of its MPMs (147T, 590S, 591R, 271A, 526R and 588T/I) were successfully accumulated to E627K (Table S8)^45^. Therefore, the type of early MPMs may be important.

Previously, MVV mutation was speculated to be the minimum essential for avian PB2 genes to acquire additional mutations for mammalian adaptation^29^. The PB2 gene of A(H1N1) pdm1918 (A/Brevig Mission/1/1918) possessed only MVV, E627K, A199S, and K702R mutations, and they may be minimum essential PB2 mutations for catastrophic pandemics^29,49^. Although A(H1N1) pdm2009 viruses without strong PB2 mutations became pandemic, the low mouse pathogenicity of SIB-5-1 is coincident with the low pathogenicity of A(H1N1) pdm2009 viruses^50^. Early mutations in the 627 domain, e.g., G590S and E627K, may be crucial for viruses to become pandemic. A strong E627K mutation may be enough for viral fitness, but a weak G590S mutation may need to recruit more mutations, Q591R and A588T, in the 627 domain^51^. Although the D701N mutation is strong, it may be unsuccessful for recruiting additional mutations to become pandemic at present. However, PB2 genes possessing I147T-K339T mutations have evolved by acquiring E627K or A588T mutation, and H5N1 viruses carried those PB2 genes^23^. The high prevalence of I147T-K339T mutations in the avian category may mean they did not mitigate viral fitness in avian hosts, and they may need to be monitored for additional mutation acquisition and mammalian transmission (Fig. 4).

In this study, we collectively compared individual and combined MPMs in the same genetic backgrounds and determined their rank orders of effects on polymerase activity, replication efficiency in mammalian hosts and mouse pathogenicity. In addition, we provided hypothetical mutational steps of avian PB2 genes during mammalian adaptation. Thus, our data and the prototypic PB2 gene may be useful to predict present and future risks and to understand the evolution of avian and mammalian PB2 genes. Additionally, our present study may encourage more systematic studies to simulate co-evolution and epistatic interaction of PB2, PB1 and PA genes of IAVs.

## Materials and Methods

### Viruses, eggs, cells and plasmids

A/Puerto Rico/8/34 (H1N1) (PR8) and PR8-derived viruses containing the mutated 01310 PB2 gene were generated using a Hoffmann’s reverse genetic system^29,52,53^. They were passaged two times in 10-day-old SPF embryonated chicken eggs (ECEs) via the allantoic cavity route (Charles River Laboratories, Wilmington, MA, USA). 293T, MDCK, and A549 cells were purchased from the Korean Collection for Type Cultures (KCTC, Daejeon, Korea). 293T and MDCK cells were maintained in DMEM supplemented with 10% FBS (Life Technologies Co., Carlsbad, CA, USA), and A549 cells were maintained in DMEM/F12 supplemented with 10% FBS. To generate recombinant viruses, Hoffmann’s reverse genetic system was used as described previously^52,53^. Recombinant viruses were generated and passaged two times in 10-day-old SPF ECEs and then stored at −80 °C until experimental use.

### Data mining and sequence analysis

PB2 genes containing a complete coding sequence were collected from the NCBI Influenza Virus Resource (http://www.fludb.org), and they were translated and compared with the BioEdit program (v7.2.5). The profiles of MPM genes were analyzed by median joining analysis with Network 5.0.0.3^54^.

### Cloning and site-directed mutagenesis

The 01310PB2 segment previously cloned into Hoffmann’s bidirectional transcription vector pHW2000 was used in this study^29^. The insert sequence was confirmed by sequencing with primers cmv-SF and bGH-SR as previously described (Macrogen Co, Seoul, Korea)^55^. Site-directed mutagenesis to introduce individual or combined MPMs into 01310 PB2 gene was performed using a Muta-direct Site Directed Mutagenesis Kit as per the manufacturer’s protocol (iNtRON, Sungnam, Korea).

### Mini-genome assay

To evaluate the polymerase activity of mutated PB2 genes, we used the pHW-NP-Luc plasmid as previously described^29^. Briefly, 293T cells in 12-well plates were cotransfected with 0.1 *μg* each of pHW-NP-Luc and mutated 01310 PB2 and the PR8 PB1, PA and NP genes. Additionally, 0.1 *μg* of the *Renilla* luciferase plasmid pRL-TK (Promega, Madison, WI, USA) was also cotransfected, which served as an internal control to normalize variations in transfection efficiency and sample processing. Then, 24 hours after transfection, luminescence was measured using a Dual-Glo Luciferase Assay System (Promega, Madison, WI, USA) in accordance with the manufacturer’s instructions on a TECAN Infinite 200 pro machine (Tecan Benelux bv, Giessen, Netherlands). All results shown are the average of triplicate experiments, and the standard deviation was calculated.

## Generation of recombinant viruses by reverse genetics

PR8-derived recombinant virus was generated by transfecting Hoffmann’s eight reverse genetics plasmids into 293T cells as described previously, with some modifications^52,53,55^. One day before the transfection, 293T cells were cultured in 6-well plates (5 × 10^5^ cells/well), and 300 ng of each plasmid was transfected together into the cells using Lipofectamine Plus reagent (Life Technologies) in 1 mL of Opti-MEM (Life Technologies). After overnight incubation, 1 mL of Opti-MEM and 0.5 µg/mL L-1-tosylamide-2-phenylethyl chloromethyl ketone (TPCK)-treated trypsin (Sigma-Aldrich, USA) were added. After another overnight incubation, 0.2 mL of the harvested culture medium was inoculated into 10-day-old SPF ECEs via the allantoic cavity route. The allantoic fluid was harvested from inoculated ECEs after incubation at 37 °C and checked for the presence and titers of recombinant viruses using plate and 96-well hemagglutination tests with 1% (v/v) chicken red blood cells according to the WHO Manual on Animal Influenza Diagnosis and Surveillance. We verified nucleotide sequences of PB1 and PA genes of selected recombinant viruses as previous^55^.

### Titration of viruses

To estimate virus titers, each virus was serially diluted from 10^−1^ to 10^−9^ in 10-fold increments, and each dilution was injected into five 10-day-old SPF ECEs as well as inoculated onto MDCK cells. The 50% chicken embryo infectious dose (EID_50_) and 50% tissue culture infectious dose (TCID_50_) were calculated using the Spearman-Karber method^56^.

### Replication efficiency of recombinant viruses in MDCK cells

To evaluate the replication efficiency of each virus, MDCK cells (2 × 10^4^/mL) were seeded in 96-well plates (100 μL/well). After 24 hours, confluent cells were washed twice with phosphate-buffered saline (PBS). Mutant viruses (10^7^ EID_50_/0.1 mL) were 10-fold serially diluted, and 200 µL of each dilution was inoculated into each well with fresh DMEM [supplemented with 1% bovine serum albumin (BSA) (fraction V) (Roche, Basel, Switzerland), 20 mM HEPES, antibiotic-antimycotic (Thermo Fisher Scientific, Waltham, MA, USA), and 1 µg/mL TPCK-treated trypsin (Sigma-Aldrich, St. Louis, MO, USA)]. During 5 days of incubation in a CO_2_ incubator at 37 °C, supernatants were collected at 5 day-post-inoculation (dpi), and virus titers were determined as described above.

### Growth kinetics of recombinant viruses in A549 cells

A549 cells were seeded in 12-well plates. After 24 hours, confluent cells were washed twice with PBS, and 0.01 multiplicity of infection (moi) of recombinant virus was inoculated into each well with 1 mL of fresh DMEM containing 0.25 µg/mL TPCK-treated trypsin. During 3 days of incubation in a CO_2_ incubator at 37 °C, supernatant was harvested at 12, 24, 36, 48, and 72 hour-post-inoculation (hpi), and virus titers were determined as described above.

### Animal experiments

Six-week-old female BALB/c mice (KOATEC Co. Pyeongtaek, Korea) were used for a mouse pathogenicity test (BIoPOA Co., Yongin, Korea). Five BALB/c mice were intranasally inoculated with 10^6^ EID_50_/50 µL of each virus after anaesthetized intraperitoneal injection of 15 mg/kg Zoletil 50 (Virbac, Carros, France). Negative control (mock) mice were injected with the same volume of sterilized PBS. To measure the 50% mouse lethal dose (MLD_50_), four mice were anaesthetized via intraperitoneal injection of 15 mg/kg Zoletil 50 (Virbac, Carros, France) and then intranasally inoculated with 10^2^ to 10^5^ EID_50_/50 µL of each virus. Mortality and weight loss were measured for 14 days. Mice that lost more than 20% of their original weight were euthanized and recorded as dead. MLD_50_ was calculated by the Spearman-Karber method. For the measurement of virus replication in infected mouse lung, six mice from each group were injected with PBS (mock) or 10^6^ EID_50_/50 µL of mutant virus. The lungs were collected at 3 and 6 dpi and then stored at −80 °C until use. The lungs were ground using a TissueLyzer 2 (Qiagen, Hilden, G) with 5 mM stainless steel beads and a volume of PBS equal to 10% of the lung weight in suspension. Then, 10 volumes of PBS were mixed with the ground tissues. After centrifugation at 2000 × g for 10 min, the supernatants were used for viral titers, which were measured as EID_50_.

### Ethics statement

All mouse experiments were carried out at BioPOA Co. (Yongin, Korea) following a protocol that adhered to the National Institutes of Health’s Public Health Service Policy on the Humane Care and Use of Laboratory Animals. The protocol was reviewed and approved by the Institutional Animal Care and Use Committee (IACUC) of BioPOA Co. (BP-2016-006-2).

### Statistical analysis

The polymerase activity and virus titer were compared by one-way analysis-of-variance (IBM SPSS Statistics ver. 23; IBM, USA). The results were considered statistically significant if P < 0.05, P < 0.01 and P < 0.001.

## Supplementary information

Supplementary information

## Data Availability

All data generated or analysed during this study are included in this published article (and its Supplementary Information files).
