# Supplementary material for: Rank orders of mammalian pathogenicity-related PB2 mutations of avian influenza A viruses
Source: Sci Rep. 2020 Mar 24;10:5359. doi: 10.1038/s41598-020-62036-5 (PMC7093554; doi:10.1038/s41598-020-62036-5)
Supplement: Supplementary file 1 — Supplementary information [file 41598_2020_62036_MOESM1_ESM.docx]

**Rank orders of mammalian pathogenicity-related PB2 mutations of avian influenza A viruses**

Chung-Young Lee^1^, Se-Hee An^1^, Jun-Gu Choi^4^, Youn-Jeong Lee^4^, Jae-Hong Kim^1,3^,

Hyuk-Joon Kwon^2,3,5^

^1^Laboratory of Avian Diseases, ^2^Department of Farm Animal Medicine, ^3^Research Institute for Veterinary Science, College of Veterinary Medicine, Seoul National University, 08826 Seoul, Republic of Korea

^4^Avian Disease Division, Animal and Plant Quarantine Agency, 177, Hyeoksin 8-ro, Gyeongsangbuk-do, 39660, Republic of Korea

^5^Farm Animal Clinical Training and Research Center (FACTRC), GBST, Seoul National University, Kangwon-do, Republic of Korea


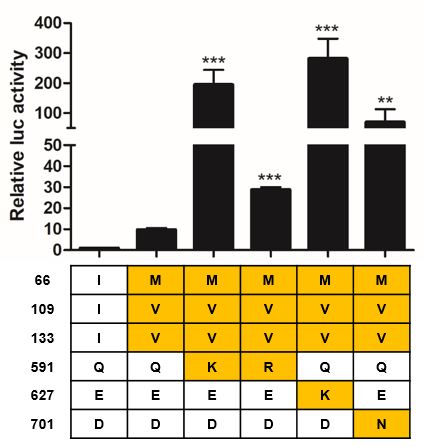


**Fig. S1. The effect of 591K/R, 627K and 701N combined with MVV mutations on polymerase activity.** Polymerase activity was measured using mini-genome assays in 293T cells at 37°C. The data were normalized to the polymerase activity of the wild-type 01310 PB2 gene. Statistical significance was calculated using one-way ANOVA (compared to PB2(01310)-MVV, ^**^*P* < 0.01, ^***^*P* < 0.001). The data are the average of three independent experiments ± s.d.

Table S1. Hypothetical mutational steps of MPMs in major genotypes of swine and human PB2 genes.

| **Name of genotype** | **The order of mutation accumulation in the PB2 gene** | | | | | | | | |
| --- | --- | --- | --- | --- | --- | --- | --- | --- | --- |
|  | 1 | 2 | 3 | 4 | 5 | 6 | 7 | 8 | 9 |
| SIB-1-1 |  |  |  | 271A |  |  |  |  |  |
| SIB-1-2 |  |  | 591R |  |  |  |  |  |  |
| SIB-1-3 |  | 147T |  |  |  |  |  |  |  |
| SIB-2-1 |  |  | 591R | 271A |  |  |  |  |  |
| SIB-2-2 |  | 147T |  | 271A |  |  |  |  |  |
| SIB-2-3 |  | 147T | 591R |  |  |  |  |  |  |
| SIB-3 |  | 147T | 591R | 271A |  |  |  |  |  |
| SIB-4 | 590S | 147T | 591R | 271A |  |  |  |  |  |
| SIB-5-1 | 590S | 147T | 591R | 271A | 588T |  |  |  |  |
| SIB-5-2 | 590S | 147T | 591R | 271A | 588I |  |  |  |  |
| SIB-6 | 590S | 147T | 591R | 271A | 588T | 526R |  |  |  |
| HIB-1-1 | 627K |  |  |  |  |  |  |  |  |
| HIB-1-2 |  |  |  |  |  | 271A |  |  |  |
| HIB-1-3 |  | 199S |  |  |  |  |  |  |  |
| HIB-2-1 | 627K |  |  |  |  | 271A |  |  |  |
| HIB-2-2 | 627K | 199S |  |  |  |  |  |  |  |
| HIB-2-3 |  | 199S |  |  |  | 271A |  |  |  |
| HIB-3 | 627K | 199S |  |  |  | 271A |  |  |  |
| HIB-4 | 627K | 199S | 702R |  |  | 271A |  |  |  |
| HIB-5 | 627K | 199S | 702R |  | 674T | 271A |  |  |  |
| HIB-6 | 627K | 199S | 702R | 9N | 674T | 271A |  |  |  |
| HIB-7 | 627K | 199S | 702R | 9N | 674T | 271A |  | 526R |  |
| HIB-8 | 627K | 199S | 702R | 9N | 674T | 271A | 588I | 526R |  |
| HIB-9-1 | 627K | 199S | 702R | 9N | 674T | 271A | 588I | 526R | 590S |
| HIB-9-2 | 627K | 199S | 702R | 9N | 674T | 271A | 588T | 526R | 590S |

Table S2. Rank orders of effectiveness of mammalian pathogenicity-related PB2 mutations on polymerase activity and replication efficiency in mammalian hosts.

| Phenotype | | Rank orders of PB2 mutations | | | | | | | | | |
| --- | --- | --- | --- | --- | --- | --- | --- | --- | --- | --- | --- |
|  |  | 1 | 2 | 3 | 4 | 5 | 6 | 7 | 8 | 9 | 10 |
| Polymerase activity | | E627K | E158G | Q591K | D253N | D701N | E192K | K526R | A588I | Q591R | T271A |
| Replication efficiency | MDCK | E627K | Q591K | E158G, D701N | | E192K, K526R, T271A, D253N | | | | A588I, Q591R | |
|  | Lung | E627K | D701N | Q591K | E158G | T271A | E192K | K526R | A588I | Q591R | D253N |

Table S3. Mutational orders of K702R, D9N, A674T, T271A, and A/V588T/I in the early human H1N1 viruses.

| Strain (accession no.) | 66M | 109V | 133V | 627K | 199S | 702R | 9N | 674T | 271A | 588T/I | 526R | 590S | 591K/R | 147T | 339T | 158G | 192K | 253N | 404L | 701N | 714R |
| --- | --- | --- | --- | --- | --- | --- | --- | --- | --- | --- | --- | --- | --- | --- | --- | --- | --- | --- | --- | --- | --- |
| A/Brevig Mission/1/1918 (ABA55038) | M | V | V | K | S | R | D | A | T | A | K | G | Q | I | K | E | E | D | F | D | S |
| A/Wilson-Smith/1933 (ABF21236) | M | V | V | K | S | R | N | P | T | V | K | G | Q | I | K | E | E | D | F | D | S |
| A/Wilson-Smith/1933 (ABD77806) | M | V | V | K | S | R | N | T | T | V | K | G | Q | I | K | E | E | D | F | D | S |
| A/United Kingdom/1/1933 (ACV49544) | M | V | V | K | S | R | N | T | T | V | K | G | Q | I | K | E | E | D | F | D | S |
| A/NWS/1934 (AFM68964) | M | V | V | K | S | R | N | T | T | V | K | G | Q | I | K | E | E | D | F | D | S |
| A/Puerto Rico/8/1934 (ABO21705) | M | V | V | K | S | K | N | T | A | I | K | G | Q | I | K | E | E | D | F | N | S |
| A/Puerto Rico/8/1934 (AGK63054) | M | V | V | K | S | K | N | T | A | I | K | G | Q | I | K | E | E | D | F | D | S |
| A/Puerto Rico/8/1934 (AGU93030) | M | V | V | K | S | K | N | T | A | I | K | G | Q | I | K | E | E | D | F | D | S |
| A/Puerto Rico/8/1934 (AAM75155) | M | V | V | K | S | K | N | T | A | I | K | G | Q | I | K | E | E | D | F | D | S |
| A/Puerto Rico/8/1934 (CAA23855) | M | V | V | K | S | R | N | T | A | I | K | G | Q | I | K | E | E | D | F | D | S |
| A/Phila/1935 (ABO38394) | M | V | V | K | S | R | N | T | A | I | K | G | Q | I | K | E | E | D | F | D | S |
| A/bh/1935 (AEM60014) | M | V | V | K | S | R | N | T | T | V | K | G | Q | I | K | E | E | D | F | D | R |
| A/Melbourne/1935 (ABD62791) | M | V | V | K | S | R | N | T | T | V | K | G | Q | I | K | E | E | D | F | D | R |
| A/Henry/1936 1936 (ABO38361) | M | V | V | K | S | R | N | T | A | I | K | G | Q | I | K | E | E | D | F | D | S |

Table S4. Earlier acquisition of E627K and A199S than K702R and D9N mutations in swine H1N1 viruses.

| H1N1 strain (accession no.) | 66M | 109V | 133V | 627K | 199S | 702R | 9N | 674T | 271A | 588 T/I | 526R | 590S | 591K/R | 147T | 339T | 158G | 192K | 253N | 404L | 701N | 714R |
| --- | --- | --- | --- | --- | --- | --- | --- | --- | --- | --- | --- | --- | --- | --- | --- | --- | --- | --- | --- | --- | --- |
| A/swine/Ehime/1/1980 (AB434405)^a^ | T | I | V | K | S | K | D | A | T | A | K | G | Q | I | K | E | E | D | F | D | S |
| A/swine/Kansas/3024/1987 (CY025017) | T | I | V | K | S | K | D | A | T | A | K | G | Q | I | K | E | E | D | F | D | S |
| A/swine/Kansas/3228/1987 (CY022476) | T | I | V | K | S | K | D | A | T | A | K | G | Q | I | K | E | E | D | F | D | S |
| A/swine/Wisconsin/1915/1988 (CY022436) | T | I | V | K | S | K | D | A | T | A | K | G | Q | I | K | E | E | D | F | D | S |
| A/swine/Wisconsin/1915/1988 (CY039924) | T | I | V | K | S | K | D | A | T | A | K | G | Q | I | K | E | E | D | F | D | S |
| A/swine/Iowa/17672/1988 (CY022340) | T | I | V | K | S | K | D | A | T | A | K | G | Q | I | K | E | E | D | F | D | S |
| A/swine/Iowa/31483/1988 (CY022977) | T | I | V | K | S | K | D | A | T | A | K | G | Q | I | K | E | E | D | F | D | S |
| A/Swine/Indiana/1726/1988 (CY039932) | T | I | V | K | S | K | D | A | T | A | K | G | Q | I | K | E | E | D | F | D | S |
| A/swine/Iowa/3421/1990 (CY096882) | T | I | V | K | S | K | D | A | T | A | K | G | Q | I | K | E | E | D | F | D | S |
| A/swine/Memphis/1/1990 (CY035077) | T | I | V | K | S | K | D | A | T | A | K | G | Q | I | K | E | E | D | F | D | S |
| A/swine/Beijing/156/1991 (KF057096) | T | I | V | K | S | K | D | A | T | A | K | G | Q | I | K | E | E | D | F | D | S |
| A/swine/California/T9001707/1991 (CY028787) | T | I | V | K | S | K | D | A | T | A | K | G | Q | I | K | E | E | D | F | D | S |
| A/swine/Iowa/24297/1991 (CY027162) | T | I | V | K | S | K | D | A | T | A | K | G | Q | I | K | E | E | D | F | D | S |
| A/swine/Maryland/23239/1991 (CY022484) | T | I | V | K | S | K | D | A | T | A | K | G | Q | I | K | E | E | D | F | D | S |
| A/swine/Beijing/216/1992 (KF057097) | T | I | V | K | S | K | D | A | T | A | K | G | Q | I | K | E | E | D | F | D | S |
| A/swine/Hong_Kong/103/1993 (GQ229281) | T | I | V | K | S | K | D | A | T | A | K | G | Q | I | K | E | E | D | F | D | S |

^a^ H1N2

Table S5. Indistinguishable mutational orders of K702R and D9N in the early strains of swine H1N1 viruses.

| H1N1 strain (accession no.) | 66M | 109V | 133V | 627K | 199S | 702R | 9N | 674T | 271A | 588T/I | 526R | 590S | 591K/R | 147T | 339T | 158G | 192K | 253N | 404L | 701N | 714R |
| --- | --- | --- | --- | --- | --- | --- | --- | --- | --- | --- | --- | --- | --- | --- | --- | --- | --- | --- | --- | --- | --- |
| A/Swine/Iowa/15/1930 (M73515) | T | V | V | K | S | R | D | A | T | A | K | G | Q | I | K | E | E | D | F | D | S |
| A/swine/JY2/1931 (CY147413) | T | V | V | K | S | R | D | A | T | A | K | G | Q | I | K | E | E | D | F | D | S |
| A/swine/USA/1976_MA/1931 CY045755 | T | V | V | K | S | R | D | A | T | A | K | G | Q | I | K | E | E | D | F | D | S |
| A/swine/USA/1976/1931 CY045747 | T | V | V | K | S | R | D | A | T | A | K | G | Q | I | K | E | E | D | F | D | S |
| A/swine/1931 CY009635 | T | V | V | K | S | R | D | A | T | A | K | G | Q | I | K | E | E | D | F | D | S |
| A/swine/Ohio/23/1935 CY027298 | T | V | V | K | S | R | D | A | T | A | K | G | Q | I | K | E | E | D | F | D | R |
| A/swine/Minnesota/24/1975 CY024940 | T | I | V | K | S | K | N | A | T | A | K | G | Q | I | K | E | E | D | F | D | S |
| A/swine/Wisconsin/11/1976 CY178718 | T | I | V | K | S | K | N | A | T | A | K | G | Q | I | K | E | E | D | F | D | S |
| A/swine/Iowa/4/1976 CY022108 | T | I | V | K | S | K | N | A | T | A | K | G | Q | I | K | E | E | D | F | D | S |
| A/swine/Kentucky/1/1976 CY022356 | T | I | V | K | S | K | N | A | T | A | K | G | Q | I | K | E | E | D | F | D | S |
| A/swine/Tennessee/7/1976 CY022044 | T | I | V | K | S | K | N | A | T | A | K | G | Q | I | K | E | E | D | F | D | S |
| A/swine/Nebraska/123/1977 CY022380 | T | I | V | K | S | K | N | A | T | A | K | G | Q | I | K | E | E | D | F | D | S |
| A/swine/Niigata/1/1977 AB434397 | T | I | V | K | S | K | N | A | T | A | K | G | Q | I | K | E | E | D | F | D | S |
| A/swine/Hong_Kong/26/1977 CY084494 | T | I | V | K | S | K | N | A | T | A | K | G | Q | I | K | E | E | D | F | D | S |
| A/swine/Kyoto/3/1979 AB434381 | T | I | V | K | S | K | N | A | T | A | K | G | Q | I | K | E | E | D | F | D | S |

Table S6. Amino acid sequence of 588 residues of incomplete mutant PB2 of human H3N2 viruses.

| H3N2 strain (accession no.) | 66M | 109V | 133V | 627K | 199S | 702R | 9N | 674T | 271A | 588T/I | 526R | 590S | 591K/R | 147T | 339T | 158G | 192K | 253N | 404L | 701N | 714R |
| --- | --- | --- | --- | --- | --- | --- | --- | --- | --- | --- | --- | --- | --- | --- | --- | --- | --- | --- | --- | --- | --- |
| A/Singapore/C2011.518/2011 (AGN50916) | M | V | V | K | S | R | N | T | A | V | R | S | Q | I | K | E | E | D | F | D | S |
| A/Santiago/p4d0/2012 (AIE53877) | M | V | V | K | S | R | N | T | A | V | R | S | Q | I | K | E | E | D | F | D | S |
| A/Boston/DOA2-101/2012 (AGV99060) | M | V | V | K | S | R | S | T | A | V | R | S | Q | I | K | E | E | D | F | D | S |
| A/Boston/YGA_01109/2012 (AHL97239) | M | V | V | K | S | R | N | T | A | V | R | S | Q | I | K | E | E | D | F | D | S |
| A/Nicaragua/7828_01/2013 (AIL94329) | M | V | V | K | S | R | N | T | A | V | R | S | Q | I | K | E | E | D | F | D | S |
| A/Montana/20/2014 (ALH30215) | M | V | V | K | S | R | N | T | A | V | R | S | Q | I | K | E | E | D | F | D | S |
| A/Pennsylvania/65/2014 (ALH30965) | M | V | V | K | S | R | N | T | A | V | R | S | Q | I | K | E | E | D | F | D | S |
| A/North Carolina/28/2014 (ALH29748) | M | V | V | K | S | R | N | T | A | V | R | S | Q | I | K | E | E | D | F | D | S |
| A/Idaho/04/2014 (ALH29518) | M | V | V | K | S | R | N | T | A | V | R | S | Q | I | K | E | E | D | F | D | S |
| A/Montana/26/2014 (ALH31466) | M | V | V | K | S | R | N | T | A | V | R | S | Q | I | K | E | E | D | F | D | S |
| A/Washington/60/2014 (ALH32803) | M | V | V | K | S | R | N | T | A | V | R | S | Q | I | K | E | E | D | F | D | S |
| A/Washington/52/2014 (ALH32916) | M | V | V | K | S | R | N | T | A | V | R | S | Q | I | K | E | E | D | F | D | S |
| A/Texas/43/2014 (ALH27559) | M | V | V | K | S | R | N | T | A | V | R | S | Q | I | K | E | E | D | F | D | S |
| A/Washington/53/2014 (ALH32314) | M | V | V | K | S | R | N | T | A | V | R | S | Q | I | K | E | E | D | F | D | S |
| A/New Hampshire/16/2015 (ALH36486) | M | V | V | K | S | R | N | T | A | V | R | S | Q | I | K | E | E | D | F | D | S |
| A/Pennsylvania/01/2015 (ALH33484) | M | V | V | K | S | R | N | T | A | V | R | S | Q | I | K | E | E | D | F | D | S |
| A/Florida/09/2015 (ALH33641) | M | V | V | K | S | R | N | T | A | V | R | S | Q | I | K | E | E | D | F | D | S |
| A/Pennsylvania/09/2015 (ALH33531) | M | V | V | K | S | R | N | T | A | V | R | S | Q | I | K | E | E | D | F | D | S |
| A/Pennsylvania/10/2015 (ALH33225) | M | V | V | K | S | R | N | T | A | V | R | S | Q | I | K | E | E | D | F | D | S |
| A/New York/06/2015 (ALH33771) | M | V | V | K | S | R | N | T | A | V | R | S | Q | I | K | E | E | D | F | D | S |
| A/Maine/18/2015 (ALH35878) | M | V | V | K | S | R | N | T | A | A | R | S | Q | I | K | E | E | D | F | D | S |

Table S7. Early strains of pre-HIB-8, HIB-8, HIB-9-1, and HIB-9-2 genotypes.

| Genotype | H3N2 Strain (accession no.) | 66M | 109V | 133V | 627K | 199S | 702R | 9N | 674T | 271A | 588T/I | 526R | 590S | 591K/R | 147T | 339T | 158G | 192K | 253N | 404L | 701N | 714R |
| --- | --- | --- | --- | --- | --- | --- | --- | --- | --- | --- | --- | --- | --- | --- | --- | --- | --- | --- | --- | --- | --- | --- |
| Pre-HIB-8 | A/Hong Kong/1/1968 (AAK51712) | M | V | V | K | S | R | N | T | A | I | K | G | Q | I | K | E | E | D | F | D | S |
|  | A/Hong Kong/16/1968 (AAO46493) | M | V | V | K | S | R | N | T | A | I | K | G | Q | I | K | E | E | D | F | D | S |
|  | A/Aichi/2/1968 (AFM71867) | M | V | V | K | S | R | N | T | A | I | K | G | Q | I | K | E | E | D | F | D | S |
|  | A/Bilthoven/15793/1968 (AFG71875) | M | V | V | K | S | R | N | T | A | I | K | G | Q | I | K | E | E | D | F | D | S |
|  | A/nt/60/1968 (AAA43613) | M | V | V | K | S | R | N | T | A | I | K | G | Q | I | K | E | E | D | F | D | S |
|  | A/Albany/17/1968 (ABO52367) | M | V | V | K | S | R | N | T | A | I | K | G | Q | I | K | E | E | D | F | D | S |
|  | A/USSR/039/1968 (AAO46495) | M | V | V | K | S | R | N | T | A | I | K | G | Q | I | K | E | E | D | F | D | S |
| HIB-8 | A/Bilthoven/21801/1971 (AFG98851) | M | V | V | K | S | R | N | T | A | I | R | G | Q | I | K | E | E | D | F | D | S |
|  | A/Memphis/3/1971 (ABP49194) | M | V | V | K | S | R | N | T | A | I | R | G | Q | I | K | E | E | D | F | D | S |
|  | A/Guandong/243/1972 (ABC67575) | M | V | V | K | S | R | N | T | A | I | R | G | Q | I | K | E | E | D | F | D | S |
| HIB-9-1 | A/Malaysia/23606/2002 (AFJ77747) | M | V | V | K | S | R | N | T | A | I | R | S | Q | I | K | E | E | D | F | D | S |
|  | A/Fujian/411/2002 (AFD64219) | M | V | V | K | S | R | N | T | A | I | R | S | Q | I | K | E | E | D | F | D | S |
|  | A/New York/485/2003 (ABB03122) | M | V | V | K | S | R | N | T | A | I | R | S | Q | I | K | E | E | D | F | D | S |
| HIB-9-2 | A/New York/1018/2006 (AHL89209) | M | V | V | K | S | R | N | T | A | T | R | S | Q | I | K | E | E | D | F | D | S |
|  | A/Netherlands/034/2010 (AFH00834) | M | V | V | K | S | R | N | T | A | T | R | S | Q | I | K | E | E | D | F | D | S |
|  | A/Singapore/H2013.751/2013 (AIG46235) | M | V | V | K | S | R | N | T | A | T | R | S | Q | I | K | E | E | D | F | D | S |
|  | A/Hawaii/63/2014 (ALH32988) | M | V | V | K | S | R | N | T | A | T | R | S | Q | I | K | E | E | D | F | D | S |
|  | A/Vermont/11/2014 (ALH32737) | M | V | V | K | S | R | N | T | A | T | R | S | Q | I | K | E | E | D | F | D | S |
|  | A/South Carolina/01/2015 (ALH33911) | M | V | V | K | S | R | N | T | A | T | R | S | Q | I | K | E | E | D | F | D | S |
|  | A/Rhode Island/06/2015 (ALH33967) | M | V | V | K | S | R | N | T | A | T | R | S | Q | I | K | E | E | D | F | D | S |

Table S8. Further evolution of HIB-9-1 and HIB-9-2 by acquiring I147T, K339T, Q591R, and D701N.

| Genotype | H3N2 strain (accession no.) | 66M | 109V | 133V | 627K | 199S | 702R | 9N | 674T | 271A | 588T/I | 526R | 590S | 591K/R | 147T | 339T | 158G | 192K | 253N | 404L | 701N | 714S |
| --- | --- | --- | --- | --- | --- | --- | --- | --- | --- | --- | --- | --- | --- | --- | --- | --- | --- | --- | --- | --- | --- | --- |
| HIB-9-1 | A/Hanoi/BM766/2003 (AEX35251) | M | V | V | K | S | R | N | T | A | I | R | S | Q | T | K | E | E | D | F | D | S |
|  | A/Peru/PER258/2010 (AHC61054) | M | V | V | K | S | R | N | T | A | I | R | S | R | I | K | E | E | D | F | D | S |
|  | A/Helsinki/824/2013 (AHA98488) | M | V | V | K | S | R | N | T | A | I | R | S | R | I | K | E | E | D | F | D | S |
|  | A/TW/872/2002 (ABD59808) | M | V | V | K | S | R | N | T | A | I | R | S | Q | T | K | E | E | D | F | D | S |
|  | A/Malaysia/2205714/2009 (AFJ78297) | M | V | V | K | S | R | N | T | A | I | R | S | Q | T | K | E | E | D | F | D | S |
|  | A/Boston/YGA_01174/2013 (AHL97996) | M | V | V | K | S | R | N | T | A | I | R | S | Q | T | K | E | E | D | F | D | S |
|  | A/California/VRDL158/2009 (ADI75938) | M | V | V | K | S | R | N | T | A | I | R | S | Q | T | K | E | E | D | F | D | S |
|  | A/Nicaragua/AGA2-18/2012 (AHZ35751) | M | V | V | K | S | R | N | T | A | I | R | S | Q | T | K | E | E | D | F | D | S |
|  | A/Rhode Island/22/2014 (ALH30622) | M | V | V | K | S | R | N | T | A | I | R | S | Q | T | K | E | E | D | F | D | S |
|  | A/Houston/JMM_146/2013 (AHZ40757) | M | V | V | K | S | R | N | T | A | I | R | S | Q | I | T | E | E | D | F | D | S |
| HIB-9-2 | A/Texas/48/2014 (ALH27952) | M | V | V | K | S | R | N | T | A | T | R | S | Q | I | K | E | E | D | F | N | S |
|  | A/Maryland/28/2014 (ALH28660) | M | V | V | K | S | R | N | T | A | T | R | S | Q | T | K | E | E | D | F | D | S |
